# Structure and function of the membrane microdomains in osteoclasts

**DOI:** 10.1038/s41413-023-00294-5

**Published:** 2023-11-21

**Authors:** Jialong Hou, Jian Liu, Zhixian Huang, Yining Wang, Hanbing Yao, Zhenxin Hu, Chengge Shi, Jiake Xu, Qingqing Wang

**Affiliations:** 1 Department of Orthopaedic Surgery, Sir Run Run Shaw Hospital, Zhejiang University School of Medicine, Hangzhou, China; 2 Department of Orthopaedics, The First Affiliated Hospital of Wenzhou Medical University, Wenzhou, China; 3 Department of Spine Surgery, Peking University Fourth School of Clinical Medicine, Beijing, China; 4 School of Biomedical Sciences, The University of Western Australia, Perth, WA Australia; 5 Shenzhen Institute of Advanced Technology, Chinese Academy of Sciences, Shenzhen, China

**Keywords:** Bone, Osteoporosis, Osteoporosis

## Abstract

The cell membrane structure is closely related to the occurrence and progression of many metabolic bone diseases observed in the clinic and is an important target to the development of therapeutic strategies for these diseases. Strong experimental evidence supports the existence of membrane microdomains in osteoclasts (OCs). However, the potential membrane microdomains and the crucial mechanisms underlying their roles in OCs have not been fully characterized. Membrane microdomain components, such as scaffolding proteins and the actin cytoskeleton, as well as the roles of individual membrane proteins, need to be elucidated. In this review, we discuss the compositions and critical functions of membrane microdomains that determine the biological behavior of OCs through the three main stages of the OC life cycle.

## Introduction

Many metabolic bone diseases, including osteoporosis,^1^ rickets,^2^ osteonecrosis,^3^ rheumatoid arthritis,^4^ and ankylosing spondylitis,^5^ are closely related to abnormal osteoclasts (OCs), which exhibit altered cell membrane structure. These different pathological diseases share commonalities, including overactivated, polarized OCs with structured membranes. Additionally, OC membrane structures are targets of many commonly used drugs. Denosumab, used to treat osteoporosis, neutralizes RANKL activity and blocks the recruitment of c-SRC, TRAF2, and TRAF6 by blocking its interaction with the receptor membrane protein RANK, thereby inhibiting the assembly of membrane protein–lipid complexes,^6,7^ whereas bisphosphonates regulate actin cytoskeleton remodeling by modulating cell membrane-mediated endocytosis.^8,9^ Thus, a detailed understanding of these membrane structures in OCs and their roles in disease progression has become a focal point in the treatment of metabolic bone diseases.

However, the study of cell membrane microdomains is currently limited. In the past, the concept of “lipid rafts” was commonly used to explain the membrane structure and behavior of OCs, which helped us to partially answer the question of how OCs respond to external stimuli.^10^ However, we found that an increasing number of OC phenotypes, such as that associated with highly proteolytic structures on OC membranes,^11,12^ are difficult to explain by lipid rafts alone. Furthermore, previous studies focused on how membrane proteins affect the morphology and differentiation of OCs, neglecting the overall role of membrane microdomains (see Table 1). Therefore, herein, we introduce the concept of membrane microstructural domains into the study of OCs. Membrane microdomains differ from lipid rafts in the following ways: (1) Membrane microdomains are complex structures comprising scaffolding proteins, and (2) they are not confined to the plasma membrane but exist throughout the cellular biomatrix system, which is discussed further in the next part.

**Table 1:** OC membrane-related proteins involved in OC membrane transport, migration, fusion, and signal transduction

Core Protein	Location	Function	OC function	Reference
SNX10	Endosomal membrane	Protein sorting	Membrane trafficking	^117^
SCL family members	Membrane surface	Transport of basic biological substrates		^118^
Myo2a	Membrane surface, F-actin?	Formation of a zipper-like structures (ZLSs) and parallel arrangements during fusion to bring the cells close to the object to be fused	Fusion	^119^
DC-STAMP	Membrane surface	Transport of the nucleus to another cell		^77^
CD47	Membrane surface	Mediation of the fusion of OC precursor monocytes through extensive contact surface contacts between chaperone cell membranes		^77,120^
Syncytin-1	Membrane surface	Mediation of the fusion of multinucleated OCs via phagocytic goblet structures		^77,120^
αvβ3	Membrane surface	Postactivation promotion of OC migration and enhanced bone resorption	Migration	^121^
Rho GTPase	Cell membrane	Promotion of cell retraction and participation in integrin-mediated signaling events		^121,122^
PLCγ2	F-actin	Regulation of integrin expression; required for the localization of Src to an actin loop		^123^
M-CSF	Cell membrane	Formation of a transmembrane complex by binding with the receptor c-Fms, forming an effective chemotactic stimulus		^44^
CSF-1R (c-fms)	Membrane surface	Receptor of M-CSF; critical OC pathway regulation of osteoclastogenesis	Signal transduction	^45^
CD36	Cell membrane	Regulation of NO signaling and the TSP-1/CD47/CD36 signaling axis		^124^

As revealed via mechanistic studies, membrane proteins affect OC differentiation and function by participating in membrane transport, migration, fusion, and signal transduction (see Table 1). The endocytosis and transcytosis of bone matrix degradation products are dependent on membrane proteins for vesicle formation.^13,14^ However, the formation of membrane microdomains and their relationships with membrane proteins in OCs remain to be further identified. Gaining an understanding of membrane microdomain formation might promote the development of OC membrane-targeted therapies. For example, membrane components extracted from bone marrow-derived macrophages (BMMs) or other OC lineages have been used in encapsulating nanoparticles, which can be used to perform targeted delivery of nanodecoys and circRNAs, potential therapeutic approaches to osteoporosis.^15,16^ Understanding how membrane microdomains engage with nanomaterials and modulate membrane curvature or form vesicles from OCs will help to reveal the specific mechanisms underlying OC differentiation and function and thus will provide a reference for therapies targeting OC membranes.^17,18^

**Fig. 1:**
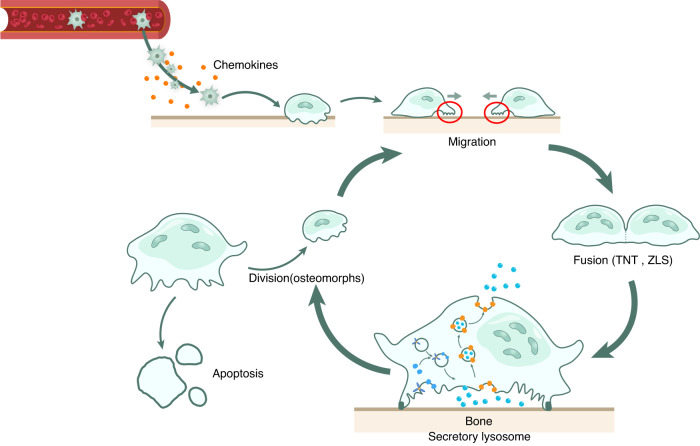
The life cycle of OCs. The life cycle of OCs is divided into three phases: (1) hematopoietic stem cells and erythroid-myeloid precursors extend filopodia from their membrane and migrate to the bone matrix;^32,128^ (2) monocytes form mature OCs (mOCs) through membrane fusion;^129^ and (3) mOCs usually continue to be multinucleated and release secretory lysosomes that degrade the bone matrix.^61,89^ In these three phases, special membrane structures are required to mediate OC differentiation and function, from migration to fusion and the release of secretory lysosomes

Therefore, the membrane microdomains in OCs need to be more deeply studied. Here, we investigate the potential role of membrane microdomains in the life cycle of OCs, including the OC migration, fusion, and maturation phases (Fig. 1).

## Membrane structure: from lipid rafts to membrane microdomains

Lipid rafts, comprising sphingolipids, cholesterol, and proteins, helps us explain some of the biological behaviors of OCs. Lipid rafts represent a good paradigm of foreign stimulus effects on OCs.^19^ The core idea of lipid–lipid interactions in a lipid raft partially explains signal transduction in osteoclasts:^20^ RANKL stimulation induces the recruitment of TRAF6, c-Src, and DAP-12 to lipid rafts, and the inhibition of TRAIL-induced lipid raft assembly inhibits TRAF6 recruitment and RANK signaling pathway activation.^21^ TRAIL interacts with the normal lipid platform to counteract the recruitment of other proteins via this lipid–lipid interaction. Similarly, we found that several different proteins in lipid raft structures directly promote or regulate OC behavior. Stomatin embedded in a lipid raft acts as a scaffold within the membrane.^22^ When dependent on specific scaffold proteins, the formation of protein complexes establishes different membrane microdomains on the basis of lipid raft constituents: for example, caveolin-1 mediates the constriction of lipid rafts to complete endocytosis.^23^ Therefore, proteins such as stomatin and Caveolin-1 act as scaffolding platforms to mediate protein‒protein interactions that are not directly mediated by lipids, and interactions between lipids may play only a regulatory role in scaffold-related protein–protein interactions.^22,23^ Thus, the concept of membrane microstructural domains was proposed to emphasize the critical role of these core proteins and protein‒protein interactions in this membrane structure.

Moreover, in contrast to lipid rafts, which are confined to the plasma membrane, membrane microdomains can be found in other cellular membranes, such as those of the Golgi, mitochondria, and lysosomes.^24^ In conclusion, we discuss the characteristics of the membrane microdomains mentioned in the introduction: a. the free distribution and b. core role of scaffold proteins. Information about the formation and distinguishing characteristics of membrane microdomains is discussed further as a supplement to this part.

**Fig. 2:**
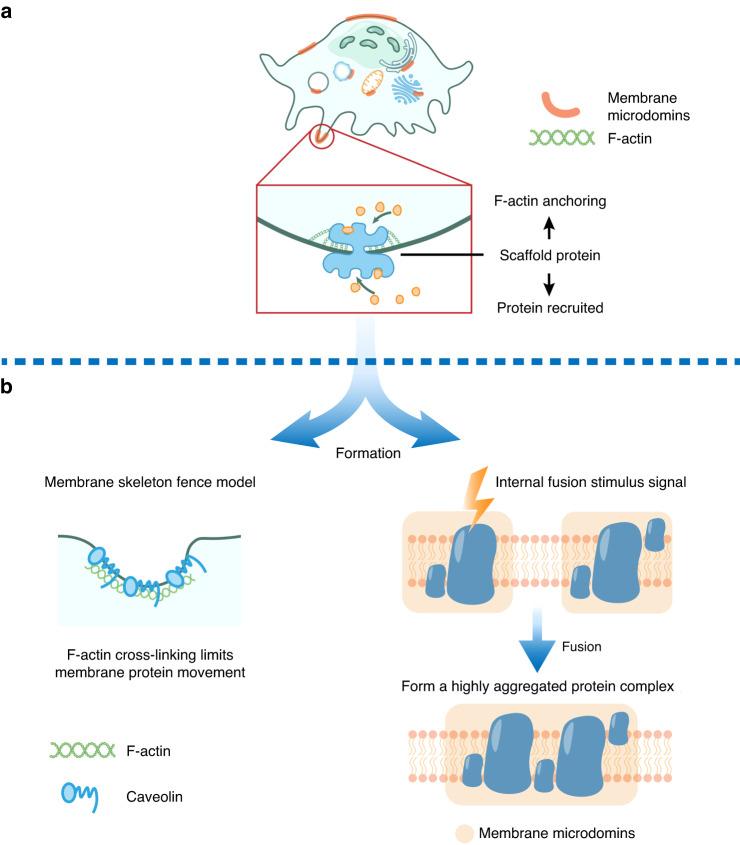
The formation and model of membrane microdomain formation in OCs. **a** Schematic diagram showing membrane structural domains in OCs. OC scaffolding proteins anchor to the cell membrane and the actin cytoskeleton and recruit proteins to form membrane microdomains. **b** Two models of membrane microdomain formation, namely, the membrane cytoskeleton fence model and the internal membrane microdomain fusion model, were proposed to provide a reference for the roles of scaffolding proteins and the cytoskeleton in membrane microdomain formation

Two models of membrane formation are proposed and shown in Fig. 2. (1) The membrane cytoskeleton fence model, which explains the formation and stabilization of membrane domains, is corresponding to protein-driven events. Some extracellular and intracellular proteins, particularly clathrins and membrane proteins, form complicated scaffolds that bind to other proteins. In addition, the membrane proteins and intracellular actin cytoskeleton are cross-linked to form a membrane cytoskeleton fence, which further anchors transmembrane proteins to the membrane and mediates the formation of a highly aggregated protein complex.^11,25^ (2) The second model involves internal fusion of membrane microdomains, where classical lipid raft structures fuse after colliding. Furthermore, membrane proteins within membrane rafts may not interact until stimulation stimulates their interaction.^26–28^

Despite the variability of membrane microstructural domains and their uniqueness in different cells of different tissues, OC can be an example to develop a general picture of the membrane microstructural domain. Additionally, some membrane microstructural domains in OCs, such as lamellipodia and tunneling nanotubes (TNTs), have been observed in other cells, and the macrostructural and microscopic scaffolding proteins among cells may share commonalities.^29–31^

Considering the summary above, we discuss our conclusions below. The membrane microdomain is a microscopic structure whose core is formed by scaffold proteins recruiting downstream proteins, crosslinking with the actin cytoskeleton after their activation and forming a general membrane-associated structure. Ultimately, the general structure may undergo macroscopic changes in membrane configuration, such as the formation of membrane tubes, pseudopodia, and ruffled borders. We temporarily named membrane microdomains with unclear scaffold proteins on the basis of their macroscopic membrane configurations, such as lamellipodia-related membrane microdomains and TNT-related membrane microdomains.

## The role of membrane microdomains in the life cycle of OCs

In this section, we focus on how membrane microdomains participate in cell migration and subsequent fusion at the OC precursor (pOC) stage and how these microdomains mediate the osteolytic and secretory functions of OCs during the mOC stage.

### Migration of pOCs: membrane microdomains serve as platforms

During OC culture in vitro, lamellipodia are often observed; they represent the direction of cell extension and are considered the hallmark structure of early OC development.^32–34^ Here, we clarify the basic functions of these membrane microstructures structures and the key proteins that constitute them.

When pOCs migrate, the structure at the leading edge of the cell dynamically extends and retracts.^35^ OCs without podosomes have spicule-like structures, which are referred to as lamellipodia, as observed after knockout of Cortactin.^36^ This structure has also been reported in the literature by Akisaka et al.^34^ The activation and formation of lamellipodia are generally believed to be induced by the Arp2/3 complex consisting of microfilamentous nucleation factors, in which the nucleation-promoting factors Wiskott–Aldrich syndrome protein (WASP) and WASP family verprolin-homologous protein (WAVE) play important roles.^34,35^ Previous findings suggest that lamellipodia are actin-based structures and that their formation depends on regulatory factors.

Lamellipodia are common in migratory cells such as fibroblasts, and their general characteristics include the broad protrusion of the leading edge and an edge that can roll back from the membrane ruffle.^37,38^ Whether lamellipodia function similarly in fibroblasts and OCs is unclear. Through the use of transmission electron microscopy (TEM), Domon T et al.^33^ showed that the morphology of migrating OCs was irregular and flat, and they confirmed that these cells have lamellipodia, indicating that via this migratory structure, OCs can move on dentin.^33^ Mature osteoclasts (mOCs) also appear to have lamellipodia on their membrane. mOCs cultured in vitro exhibited stretched out lamellipodia that can mechanically decompose substrates and bring the substrates to the surface of the cell body via retraction of the lamellipodia.^39^ Although lamellipodium-like structures were observed in this study, the specific differences between lamellipodia in the pOC and mOC stages remain unclear. Notably, we initially focus our attention on lamellipodia during the migratory phase.

Lamellipodia determine the direction of cell migration, and lamellipodium stretching requires the actin network; therefore, we need to identify the scaffolding and regulatory proteins that determine the OC membrane structure.^40^ Structurally, focal adhesion anchors the cell to the matrix and thus provides the mechanical force needed for actin contraction.^37^ Additionally, cells migrate not only by extending lamellipodia, which are formed by scaffolds via an actin network but also by extending filopodia, which contain only part of the actin bundle and aggregate to form lamellipodia.^39,41^ Hence, matrix anchoring and actin movement are the two critical components of pseudopod formation and movement.^37^ We conclude that matrix anchoring is dependent mainly on membrane proteins to form the first structural platform, while the actin that is recruited forms the second platform and mediates contractile motility based on regulatory factors to establish actin flow.^37,42,43^

**Fig. 3:**
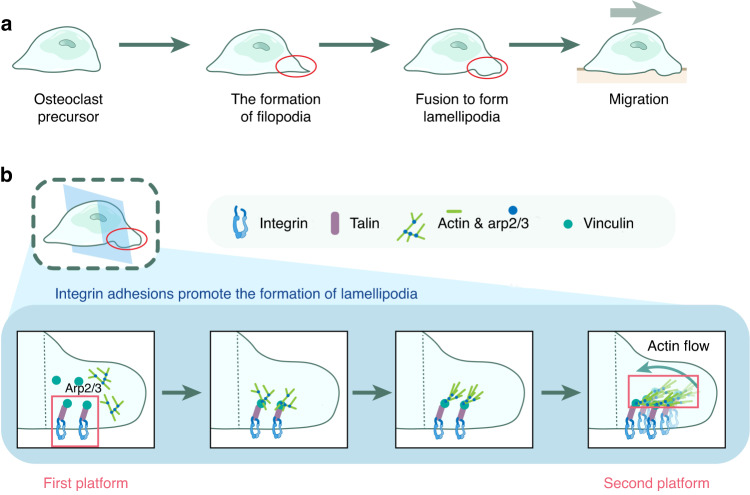
Lamellipodia and their formation. **a** Schematic diagram at the macroscopic level: the process of lamellipodium formation. pOCs form filamentous pseudopodia, and their fusion drives lamellipodium formation, which determines the direction of cell migration. **b** Schematic diagram at the microscopic level: the process of integrin adhesion promoting lamellipodium formation. Longitudinal sections of lamellipodia show that integrins recruit the regulatory proteins talin and vinculin, which regulate actin skeleton remodeling mediated via Arp2/3 to initiate reverse actin flow and mediate pseudopod contraction on the basis of the counteracting force provided by the integrin adhesion bodies. In this process, integrins and regulatory proteins form the scaffolds of the pseudopod membrane microdomains and then integrate actin, leading to the formation of membrane macrostructures

In OCs, the initiation site of the lamellipodium membrane microdomain may be composed of integrins or other adhesion receptors (e.g., other receptor tyrosine kinases (RTKs) such as colony-stimulating factor 1 receptor, also known as c-FMS^44^) that activate downstream regulatory proteins, including GTPases, protein kinases, and phosphatases, to induce the ARP2/3-related actin network and thus form the “second platform”.^45,46^ Considering the work of Boujemaa–Paterski, Rajaa et al., Geiger, Benjamin et al. and Fukunaga, Tomohiro et al., we propose a model based on integrin adhesion in OCs. (1) Integrins recruit and activate vinculin via talin to form nascent adhesions. (2) Vinculin is recruited and binds highly branched F-actin networks and contracts to establish actin flow, at which point the adhesions mature. (3) Centripetal actin flow at 1–3 μm·min^−1^ may stimulate the maturation of other nascent adhesions. (4) Eventually, many adhesions accumulate, enhancing mechanical resistance and ultimately leading to expansion of the leading edge of the lamellar pseudopod (Fig. 3).^42,43,47^ Among the integrins highly expressed in OCs, αvβ3^48^ but not other integrin subunits has been shown to colocalize with vinculin, talin, and arp2/3. RTKs, such as epidermal growth factor receptor (EGFR), can also form the first platform based on its regulation of downstream PI3K, SRC, RAS, and RAC expression, and the modulation of the ARP2/3 complex and WASP affects the formation of the actin network and lamellipodia.^32,49–52^ Although evidence to support a role for RTKs in lamellipodium formation in OCs is insufficient, the inhibitory effect of RTK inhibitors on osteoclasts suggests that RTKs may be involved, which warrants further study.^53,54^ Other adhesion-related proteins, including cadherin, also remain to be investigated as regulators of lamellipodium formation.^55–57^ Notably, many lattice-like protein sheets have been found at the edge of filamentous pseudopodia.^58^ Although not colocalized with the OC actin network, this protein lattice is tightly bound to the apatite surface and may act as an adhesive rather than an endocytic agent. Therefore, it remains unclear whether this part of the protein lattice can serve as a scaffold for establishing the structural domain of the lamellar pseudopod membrane.

In conclusion, the identification of essential assembly sites in the microstructural domain of the lamellar pseudopod membrane may facilitate the development of locally acting regulators of early OC polarization. Although it remains unclear whether adhesion receptors in addition to integrins are involved in the assembly process, targeted regulation of the “first platform” and “second platform” in lamellipodium formation may facilitate the selective regulation of OC functions.

### OC fusion: membrane microdomain interactions with the actin cytoskeleton

The actin cytoskeleton of OCs is a dynamic structure that changes rapidly during cell migration, fusion, and resorption. The membrane microdomains of OCs need to be supported by the actin cytoskeleton, and when cortical actin is reconstructed, the cell membrane structure changes accordingly.

**Fig. 4:**
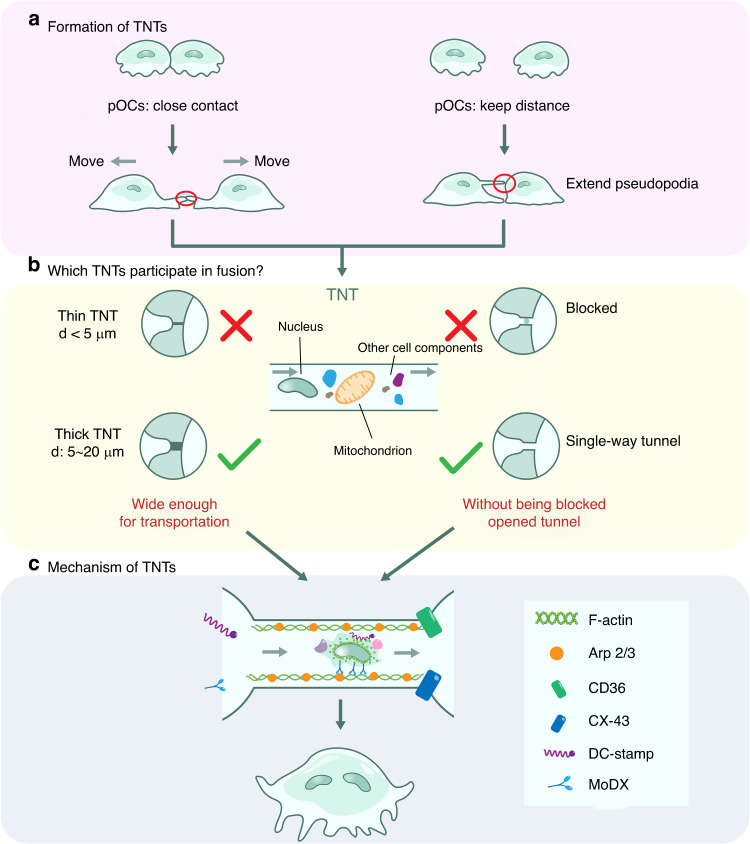
Early fusion: OCs fuse through TNTs. **a** Two mechanisms explain TNT formation: filamentous pseudopods extend between fusion partners or nearby fusion partners that have separated from each other by the action of chemokines, and a TNT is formed at the interconnection of the plasma membrane between fusion partners**. b** Nuclear translocation is possible when a TNT has (1) a diameter in the range of 5–20 µm and (2) an open interconnection inside the duct. **c** The processes and mechanisms by which membrane microdomains mediate nucleus transport

**Fig. 5:**
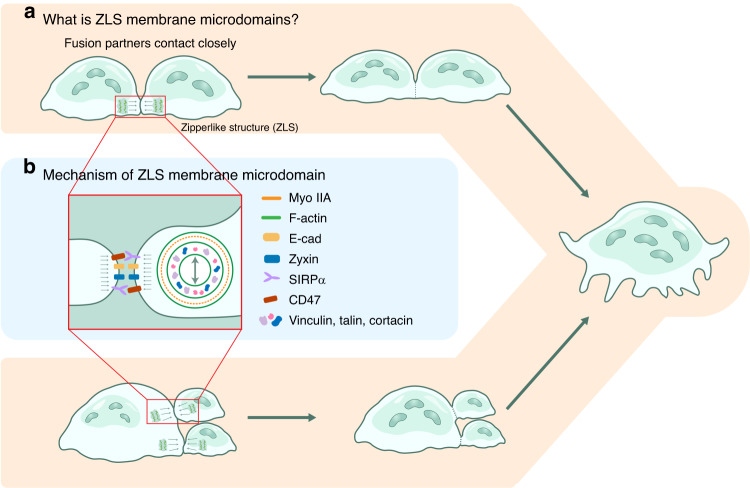
Late fusion: OCs undergo multinucleated cell–multinucleated cell and multinucleated cell–mononuclear cell fusion through ZLS structures. **a** Fusion partners are closely linked through actin flow, and the actin cytoskeleton forms a ZLS structure at a contact point. **b** The structure of the ZLS membrane microdomain, including the surface membrane proteins and the internal actin complex

During the fusion phase of the OC life cycle, the actin cytoskeleton promotes the extension of filopodia between cells or actin flow, which results in the formation of the characteristic TNT membrane domain structure (Fig. 4) or ZLS (Fig. 5) to trigger fusion.^59,60^ In the early stage of fusion, monocytes rely on their TNTs to fuse with another monocyte and thus generate multinucleated cells.^60^ The later stage is dominated by the formation of ZLSs between multinucleated cells and their fusion partners.^61^

#### TNT-associated membrane domains, not filopodia, are the keys to early fusion

The conventional view is that at the early stage of OC fusion, pOCs filopodia protrude to initiate fusion with partners. Although the role of filopodia has been demonstrated, many questions, such as how the nucleus is delivered and how filopodia trigger fusion, remain unanswered.^59,62^ Recently, a TNT, which is a very thin membrane tube, was found at the head of a filopodium where two cells contacted each other.^60^ Therefore, TNTs, not filopodia, may directly participate in the connection between fusion partners and drive material transport. TNTs are thought to be important in cell communication among bone marrow-derived cells (including macrophages, OCs, and dendritic cells) and in the fusion of macrophages and pOCs. Therefore, to clarify the role of TNT-related membrane domains in OC fusion, we need to define which TNTs can mediate fusion and the key mechanisms through which TNT-related membrane domains are involved in fusion.^31,63^

##### How do TNT-associated membrane microdomains form?

First, as described by McCoy–Simandle, Kessler et al., a TNT is identified according to the following three phenotypic criteria: (1) it connects at least two cells, (2) it contains F-actin, and (3) it does not attach to the matrix but extends from filopodia. This definition can be used to distinguish a TNT from any other F-actin-rich structure, and a TNT may be considered a special membrane microdomain.^64^ A TNT is generated in two situations: when filopodia protrude between fusion partners and when two cells located next to each other are separated under the action of chemokines.^65^ In these cases, a tube is formed in the plasma membranes where the fused cells are connected and cellular components such as organelles are transported (Fig. 4a).

##### Which TNTs participate in OC fusion?

TNTs are designated closed or open depending on whether they are connected to the target cell.^65^ Previous studies have mainly suggested that closed-end TNTs mediate gap junction formation, but after their conversion into open-ended TNTs, TNTs are known to participate in a process similar in virus‒cell membrane fusion or cell‒cell fusion.^66,67^ In addition, TNTs are classified into two functionally distinct types according to their size: (1) those with a diameter less than 5 µm are thin TNTs and contain only F-actin, and (2) those with a diameter ranging from 5 to 20 µm are thick TNTs and contain both F-actin and microtubules. Previous studies have revealed that large organelles, including lysosomes, mitochondria, and even nuclei, can be transferred only through thick TNTs (Fig. 4b).^60,65^

Therefore, although TNT-associated membrane microdomains spanning pOCs at the fusion stage have been observed,^60^ it is thought that TNTs participate in OC fusion only when their diameter is in a specific range (5–20 µm) and when there is intercommunication within the tunneling tube.

##### How do TNT-associated membrane microdomains participate in OC fusion?

M-Sec is a key factor in the formation of a TNT; its expression is upregulated during osteoclastogenesis, and M-Sec depletion significantly inhibits OC fusion by inhibiting TNT formation.^31,68^ Nonetheless, the specific mechanism through which TNT-associated membrane microdomains mediate cell fusion remains to be elucidated. pOCs recognize distant fusion partners through long intercellular F-actin structures. When two cells approach each other, thin and short actin protrusions (approximately 10 μm) can be observed on the leading edge of the cells.^69^ Nuclei have also been observed in these structures,^70^ suggesting that the nucleus may be transported through tubes formed by the actin cytoskeleton, which may trigger prophase fusion.

Based on these findings, we asked the following question: What is required for TNTs to mediate fusion? (Fig. 4c).

1. Membrane proteins, the surface proteins in the membrane domain of TNTs, can recruit the actin cytoskeleton. Two types of membrane proteins involved in TNT function have been found in Ocs: DC-STAMP,^71^ which shows transport activity, and connexins, including CD36^72^ and CX-43.^73^ Although these proteins have been identified, the components of the TNT shell have not been fully characterized, and the exact mechanisms underlying TNT functions remains unclear.
2. Actin-related regulatory proteins: Myosin is critical for providing power to F-actin and is often recruited to the membrane domain. Myosin 10 (MyoX) has been identified as a molecular motor that regulates TNT formation. This unconventional myosin is specifically expressed in OCs.^74^ As shown through in vitro experiments, pOCs remained in a monocyte state after MyoX expression was reduced by shRNA. This result was largely obtained to MyoX binding to microtubules through its MyTH4 tail domain, regulating F-actin cytoskeleton dynamics to promote the formation of an ordered TNT. Moreover, DC-STAMP, a transmembrane protein in the structural domain of the TNT membrane, penetrates other precursor cells by further interacting with the F-actin backbone to achieve migration through TNTs.^31^
3. Interactions between actin and the perinuclear cytoskeleton: The nucleus is sometimes located within the microtubule-actin network, which mediates its transport, and the microtubule–actin filaments usually originate from the perinuclear region, which suggests that the nucleus and F-actin are closely related. Moreover, some regulatory proteins play irreplaceable roles in nucleus-related F-actin dynamics. The actin-binding ARP2/3 complex stabilizes bent and branched actin structures, whereas c-Src and cortactin colocalize with F-actin at the cell periphery, which suggests that the latter may participate in the rearrangement and stabilization of bent and branched F-actin networks.^70^ In addition, c-Src, cortactin, cofilin, and actin can accumulate around the nucleus, suggesting that their involvement in nuclear movement might partially involve the regulation of nucleus delivery via thick TNTs.^70,75,76^

##### Summary

Thus, TNT-associated membrane microdomains facilitate the transport of substances, including nuclei, and this process requires signal recognition mediated by surface molecules, including DC-STAMP, and interactions between F-actin and the perinuclear cytoskeleton. However, only a fraction of the relevant proteins in a TNT have been identified, and the proposed structural domain of the TNT membrane suggests that the scaffolding proteins in this structure not only include marker proteins of intercellular connections but also bind the intracellular actin cytoskeleton to the perinuclear frame. Here, we summarize only some of the components involved in these intercellular linkages, as their specific relation to nuclear transport events via natural scaffolding proteins remains to be discovered.

#### ZLS-associated membrane microdomains are key for multinucleated cell fusion

##### What are ZLS-associated membrane microdomains?

After single-nucleated precursor cells fuse to form multinucleated cells, they still need to combine with other fusion partners to form multinucleated OCs with more than three nuclei and podosome belts.^59,61,77^ The experiments conducted by Takito, Jiro et al. revealed that the F-actin cytoskeleton of multinucleated cells agglomerates form a zipper-like F-actin structure when in contact with other multinucleated cells, which has also been shown to be the basic manner through which multinucleated cells fuse.^59,78^ Therefore, elucidation of the ZLS membrane microdomain is extremely important to clarify the life cycle of OCs.

##### Formation and function of the ZLS

A ZLS and its associated membrane microdomains have attracted our interest. Membrane proteins in a TNT may recruit actin-related regulatory proteins by downstream signaling to then associate with cortical actin in the perinuclear area. In contrast to TNT-related membrane microdomains, ZLSs appear to mediate the closeness between two precursor OCs through a complex composed of F-actin and regulatory proteins. Force may be critical in directly promoting fusion events. When mononuclear and multinuclear cells collide with actin rings, the cell membranes at the collision site move in response to actin flow, leaving the plasma membranes close together, and F-actin condenses on the plasma membrane to form a cluster of ZLSs (Fig. 5).^78^ The formation of this structure is the basis for subsequent cell membrane fusion events. Subsequently, when plasma membranes are fused via actin flow, the ZLSs are reconstituted, and cortical actin is cleaved to form a foot vesicle band. Thus, these multinucleated cells fused by ZLSs can give rise to larger OCs (Fig. 5).^59,78^ Clarifying the mechanism through which the membrane microdomain and F-actin cytoskeleton induce ZLSs will help us elucidate the key mechanisms underlying the later stages of OC fusion.

##### Motility forces of the actin cytoskeleton and ZLS-associated membrane microdomains

Published studies have not clarified how E-cadherin and integrin β3 on the surface of ZLSs regulate actin flow or stability of the F-actin cytoskeleton. Dufrançais, Ophélie et al. found that these structures are not involved in the early fusion process but may stabilize adhesion points, promote migration, or induce protein hydrolysis in the later fusion phase.^60^ In contrast, migration and adhesion between multinucleated cells and fusion partners may be facilitated by binding between membrane proteins, which induces downstream signaling and establishes actin flow. Accordingly, we focused on the potential role of the actin cytoskeleton and intracellular motility forces (Fig. 5).

1. Actin backbone: Arp2/3 and cortactin are colocalized with actin at the center of a ZLS, and the core framework of a ZLS is based on F-actin and nonmuscle myosin IIA. The periphery of the structure is covered with paxillin and vinculin to regulate its traveling wave motion.^78^ The cell contact surface is also covered with fusion-related proteins, including zyxin, E-cadherin, CD47-SIRPα, and integrin β3, forming a composite structure consisting of the intracellular cytoskeleton and plasma membrane proteins.^79,80^
2. Actin flow: Actin within a single podosome “foot” undergoes vertical oscillatory motion, which in turn forms a traveling wave. An analysis of the spatiotemporal location of podosomes revealed that the vertical motion is based on two factors, namely, regulatory protein comovement patterns (vinculin and talin show similar vertical oscillations) and actin aggregation and assembly in the podosome core.^81^ In turn, this traveling wave triggered by the overall vertical oscillation of the actin cytoskeleton moves in such a way that neighboring cells squeeze against each other. Additionally, the distribution of the F-actin bundle at the OC podosome overlaps with that of myoIIA, the activation of which leads to the generation of circumferential forces and helps maintain a balancing effect on actin wave motion.^78^

##### Summary

Similar to TNTs, ZLSs contain and regulate actin proteins such as cortactin, paxillin, and vinculin.^78^ Moreover, the ZLS membrane microdomain similarly lacks a backbone protein that integrates the membrane protein component mediating contact recognition with the actin regulator of traveling wave formation. Importantly, the mechanism through which stomatin is bound by contact partners via exosomes and further mediates contact adhesion suggests that the structural domain microfusion mechanism that we propose may be activated at this stage; this hypothesis was assessed in previous studies, and further investigation of the mechanism underlying ZLS membrane microdomain formation is needed.^22^

### Bone resorption and secretory lysosomes

The classical structural signature of mature OCs is the formation of F-actin-rich adhesion structures on the ventral membrane contacting the bone surface, i.e., the ruffled border.^1,58^ This membrane is called a ruffled border (RB) due to the large number of folds.^1,82^ Although RBs have been shown to be involved in the process of OC bone resorption, it remains unclear exactly how it functions.

**Fig. 6:**
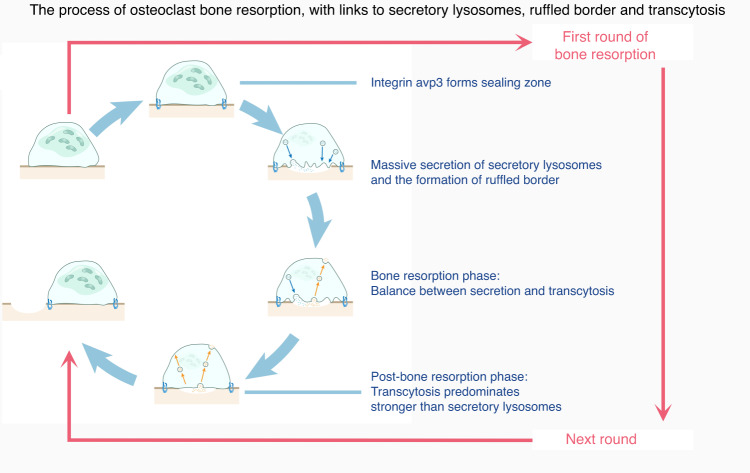
mOCs exert their osteolytic function by adopting a secretory lysosomal structure. The process of OC bone resorption is related to secretory lysosome production, the RB and transcytosis. Activation of integrin signaling during initial bone resorption leads to development of a sealing zone for OC bone resorption. Accordingly, many secreted lysosomes are fused to the plasma membrane within the sealing zone, leading to the formation of ruffles. Secretory lysosomes are secreted mainly into peripheral subdomains of RBs and in the central subdomain, which is thought to be the site of transcytosis. Endocytic vesicles are formed in the central subdomain and transported to the apical side of the cell. During bone resorption, secretory lysosomes initially play a key role in facilitating the rapid formation of RBs, whereas transcytosis depletes the ruffles and facilitates the endocytosis and secretion of osteolytic products from the bone resorption lumen to the extracellular surface. At the onset of a new cycle of bone resorption, RBs are formed and depleted again

The RB is enclosed by a sealing zone composed of integrin αvβ3 as the core protein and V-ATPase, a transporter protein (such as CLC-7), a small GTPase, and lysosome-associated membrane proteins (LAMP1, 2).^83–86^ However, Mika T K Mulari et al. showed that the RB is also divided into an uptake region and a release region.^1,85^ The release zone mediates vesicle entry into a cell that stores enzymes and acidic ions, thus allowing the release of lysosomal contents into the bone matrix enclosed by the sealed zone, followed by internalization of the osteolysis products into vesicles in the resorption zone, which are then released to the functional secretory domain (FSD) at the tip of the cell via transcytosis (Fig. 6).^87,88^ In this process, the RB acts as a “transit station”, mediating the output and input of vesicles.

Hence, we aim to elucidate the mechanism underlying RB generation and the occurrence of bone resorption by focusing on secretory vesicles.

#### Secretory vesicles

Although we currently do not know whether transcytosis is necessary for the osteolytic function of mature OCs, the output of vesicles is considered crucial for osteolysis, which is why we focus first on these secretory vesicles.^89,90^ Notably, another reason for this focus on secretory vesicles is that the RB is equivalent to a transit station that mediates secretory lysosome release and vesicle transcytosis, which means that the membrane microdomains are somewhat similar among these three structures, and therefore, it is necessary to elucidate the membrane microdomains of secretory vesicles.

In contrast to other cells, OCs have evolved specific lysosome-associated organelles (LROs), which are acidic vesicles that specifically secrete osteolytic proteins. These secretory vesicles are thus called secretory lysosomes.^91,92^ Research on the unique characteristics of the membrane microdomains of secretory lysosomes is worth considering.

Based on recent literature, we found that V-ATPase performed both scaffolding and recruitment roles in the membrane microdomain, and these functions are closely related to different V-ATPase subunits (Table 2).^93–95^ We propose that the V-ATPase a and d subunits, which recruit downstream signaling factors and affect cellular localization, are critical for V-ATPase scaffolding action.^89,96,97^

**Table 2:** The functions of different V-ATPase subunits^112,125–127^

Complex	Subunit	Function
V1	*A, B*	Mediate ATP binding and hydrolysis via heterohexamers formed by the V1A and V1B subunits to power V-ATPase function
	*E, G*	Stabilization
	*C, H*	Regulation
	*D, F*	Structural support Form a central rotor axis
V0	*a*	Cell localization
	*c*	Barrel movement
	*d*	Support for *a subunit* formation
	*e*	Binding to *a subunit*

More subunit interactions and site mutations that affect V-ATPase subunits are discussed in a previous article^127^

##### The kinetic marker and scaffolding protein V-ATPase

The membrane microdomain of secretory lysosomes in OCs has some specific features. V-ATPase is a key structure mediating lysosomal transport, and the heterogeneity of its subunits determines their distribution and subcellular locations in different cells. Knockdown of the a3 subunit inhibits its plasma membrane-targeting ability, leading to the suppression of bone resorption. Previous studies have suggested that OC bone resorption requires V-ATPase containing the a3 subunit.^95,98^ In addition, a3 subunit assembly involves assembly of the d subunit (the d2 isoform) heterodimer, which is expressed at fourfold higher levels in OCs than the d1 subunit prevalent in other cells, suggesting that V-ATPase consisting of the a3 and d2 subunits is essential for OC membrane behaviors.^89,99,100^

The membrane microdomains of secretory lysosomes in OCs are composed mainly of ATPase as the core protein because ATPase not only maintains acidity inside the lysosome but also supplies energy to enable interactions with Rab family members and regulate the Rab protein guanine nucleotide binding for targeted lysosome transport.^94,101^ This finding indicates that V-ATPase is not only a membrane marker of secretory lysosomes but also the driving force for their function (Fig. 6).

Based on our understanding of V-ATPase, we have additional questions to answer: How does V-ATPase respond to signaling that drives the transport of secretory lysosomes, and how is V-ATPase oriented relative to the RB?

In response to the first question, V-ATPase can bind to small GTPases and regulatory proteins in a vesicular pH-dependent manner, which implies that V-ATPase plays a role in not only promoting an acidic pH environment but also in transmitting acid-dependent signaling.^102^ The a and c subunits of the V0 complex may play primary roles in linking these activities. Although these mechanisms are not fully understood, we can conclude that the maturation of pH-related signaling in secretory lysosomes likely initiates the bone resorption process.

##### Movement and release: Membrane microdomain components in secretory lysosomes

**Fig. 7:**
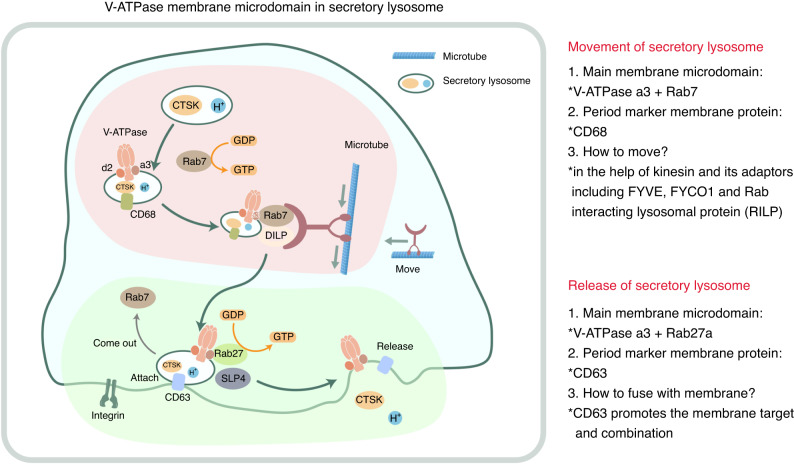
Membrane microstructural domains of V-ATPase secretory lysosomes. Rab7^GDP+^ binds to the a3 isoform of V-ATPase in the lysosomal membrane, and then, GDP is replaced by GTP. A lysosome binds to motor proteins via Rab7^GTP+^, which in turn colocalizes with the lysosomal plasma membrane via the action of CD68. Subsequently, Rab7 moves inward along microtubules in collaboration with in-adapter-Rab-interacting lysosomal protein (RILP), while FYVE encoded by FYCO1, the adapter of the kinesin driver protein, moves outward and participates in vesicle transport. Near the plasma membrane, Rab27a preferentially binds to CD63-positive secretory lysosomes, maintains their stability under the action of slp4, and then binds to the plasma membrane with CD63 as a marker, thereby mediating lysosomal content release

We explored the role of the membrane microdomains in the movement of secretory lysosomes, including membrane-bound transport and membrane-bound attachment (Fig. 7).

(I) Membrane-bound transport with CD68 as a marker and Rab7 as a motility driver
  Rab7 (the GDP-bound form) binds to the a3 isoform of V-ATPase in the lysosomal membrane.^103^ After activation, Rab7 (the GTP-bound form) binds motor proteins and their adapters to move inward along microtubules.^103,104^ CD68 is a lysosomal marker at this stage that colocalizes with lysosomes and the OC plasma membrane.^105^
(II) Membrane-bound attachment with CD63 as a marker and Rab27 as a motility driver
  Rab27 is involved in the fusion of the lysosomal membrane with the plasma membrane, and Rab27a knockdown suppresses the binding of the CD63-labeled intracellular compartment to the plasma membrane. Moreover, when Rab27a binds to GTP and is activated, the effector proteins Slp4 and Rab27a can colocalize with a structural compartment containing CD63 to enhance the stability of secretory lysosomes after binding to the plasma membrane.^106^

Furthermore, secretory lysosomes are predicted to bind to Rab7 and Rab27a through the a3 subunit of V-ATPase, which activates their downstream effectors to maintain their stability and plasma membrane attachment. Thus, the identification of Rab effectors that regulate the cell polarity-directed transport of secretory lysosomes in OCs may help clarify the specific transport patterns of secretory lysosomes.

In summary, mOCs undergo targeted transport and binding as well as show polarity through the actions of plasma membrane and the membrane microdomain of secretory lysosomes that recruit different Rab proteins with different kinetic properties as drivers for transport at different stages. Therefore, identifying the critical scaffolding proteins or complexes that integrate energy exchange factors is expected to enable the elucidation of the complete mechanism underlying secretory lysosome movement in the OC-mediated osteolysis process.

#### The ruffled border and transcytosis

Based on the aforementioned information, we conclude that RB is closely related to the vesicle cycle in OCs. On the one hand, secretory lysosomes in OCs are targeted to the plasma membrane based on the V-ATPase-associated membrane microdomains and are incorporated into it via membrane fusion. The key membrane proteins from secretory lysosomes, such as CCL, remain on the plasma membrane, expanding the membrane area, and then, the plasma membrane bends to form a ruffled border (Table 3).^1^ During the formation of a RB, small connecting bands are observed on the outer side of a forming RB, and they are gradually assembled into an RB, and therefore, the peripheral region of the ruffled border is a key “site” for membrane fusion^107^ (Table 4).

**Table 3:** Protein components common to RBs and the secretory lysosomes membrane

Sequence number	Category	Function	Protein
I	Active macromolecular membrane transport proteins	Facilitate movement	V-ATPase
II	Ion channel	Acidifies and maintains the membrane potential	CLC-7
III	Lysosomal membrane proteins	Link *secretory lysosomes*	LAMP1/2
IV	Family of membrane transporter proteins		The Rab GTPase family of proteins, SNX (sorting nexin)
V	Autophagy-related components

**Table 4:** Functions and future prospects of the main membrane microdomains in OCs

Membrane microdomain	Function	Future prospectives
Migration: Lamellipodium -related microdomain	Lamellipodia appear in both pOCs and mOCs, at least in migrating cells, and they determine the direction of cell migration. We propose that this macroscopic membrane structure may comprise microdomains of two key “platforms”: the integrin adhesion complex and the cytoskeleton network cross-linked to the membrane. Of course, in addition to integrins, the adhesosome assembly may be mediated by other receptors functioning as scaffolds. Considering membrane microdomains, if we intervene with other potential; scaffolding proteins and identify the key downstream proteins, we may be able to link the processes involved in lamellipodium-related formation and regulation.	Based on lamellar pseudopods, identification of other integrin-like scaffolding proteins may be necessary. Moreover, the mechanism underlying integrin downstream mediation of lamellipodium formation needs to be elucidated, or at least, it should be identified, as in the case of other pseudopod and peduncle structures. In terms of the functions of lamellar pseudopods, migration, and mechanical disassembly are the two major functions identified thus far, and they correspond to pOCs and mOCs, respectively. Do the same lamellipodia form in OCs in different periods? Is there a difference in the scaffolding proteins and molecular mechanisms of these two functions related to membrane microdomains?
Fusion: TNTs microdomain	TNTs are critical conduits for material exchange between OCs, and when they transport a nucleus, they initiate mononuclear-mononuclear pOC fusion. However, this process may require the mobilization of substantial intracellular resources to coordinate membrane signaling, intracellular cytoskeletal action, and perinuclear cytoskeleton formation. This series of processes is not yet clear.	At the fusion stage, involving TNTs or ZLSs, membrane microdomains have one thing in common: the endpoints triggering the signaling downstream of the membrane microdomain action are predictable, but the proteins in the microdomains and how they respond to signaling remains unclear. Moreover, membrane fusion requires mutual contact between fusion partners; does the mutual contact of membranes affect membrane microdomains (e.g., are different subdomains of scaffolding protein involved in fusion)?
Fusion: ZLS microdomain	A ZLS is crucial to the fusion of multinucleated osteoclast precursors, which ultimately establishes opposing actin flow in the membrane–membrane contact region. It acts like a fist to compress the membrane contact region, thus enabling membrane fusion. Events triggered downstream of membrane microdomain action have been experimentally confirmed, but the origin of ZLS formation at the membrane microdomain level remains to be demonstrated.	
Bone resorption: V-ATPase-related membrane microdomain in secretory lysosomes	The ruffled border is the classical hallmark of the membrane structure of mature osteoclasts. However, the ruffled border can be further divided into an absorptive subdomain and a secretory subdomain, where transcytosis and secretory lysosomal release occur, respectively. The balance between these functions affects the formation and maintenance of the ruffled border. Additionally, the membrane microdomain of secretory lysosomes, marked by V-ATPase, can bind the intracellular cytoskeleton for membrane-targeted transport. This typical secretory event is important in bone resorption by osteoclasts.	The ruffled border, secretory lysosomes and transcytosis vesicles participate together in the membrane recycling process of mOCs. Here, We describe secretory lysosomes as an entry point, but many questions remain to be answered: 1) How do secreted lysosomes form: how can V-ATPase be used as a scaffolding protein in these membrane microdomains, and how can secretory lysosomes be distinguished from phagolysosomes, endosomes, and nonsecretory lysosomes? 2) How are membrane subdomains of the ruffled border divided? How do membrane microdomains at the periphery and center mediate the secretion and uptake of vesicles? 3) Does V-ATPase also serve as a core protein for the transcytosis of vesicles derived from their membrane microdomains?

On the other hand, when OCs undergo the bone resorption process at one site, they endocytose the corresponding metabolites into a resorption pit into the center of the RB. Then, vesicles are formed and transport metabolites to the FSD at the top of the cell and release them. This process, referred to as transcytosis, is a critical mechanism of OC secretory function in bone.^85,88,90,108–111^

We discussed the key membrane domains of secretory lysosomes in the previous section, but the role of secretory vesicles in transcytosis has not been addressed. The secretory lysosomal components in an RB have been identified. Whether these components are utilized by transcytotic vesicles remains to be explored. It also remains unclear whether V-ATPase and other specific Rab molecules are crucial proteins that participate in bone secretion. These gaps in knowledge have not been explained in the literature to date, but we believe that the membrane microdomain of secretory lysosomes will provide a paradigm for further investigation into the key structures of secretory lysosomes. The process of V-ATPase vesicle formation involving actin has been described in Han, Guanghong et al.^112^

## Summary and future prospects

Given that the full range of OC biological behavior cannot be readily explained by lipid rafts, we introduce the concept of membrane microdomains in OCs and refine it based on OC-specific biological behaviors. In contrast to lipid rafts, membrane microdomains are similar to complexes that fuse with core proteins in the membrane and their associated lipids. These complexes include the cytoskeleton, which is tightly cross-linked to membrane lipids and proteins. The stimulation of membrane microdomains changes the interactions among their scaffolding proteins, resulting in a shift in the entire structure from a resting state to an activated state. Membrane microdomains are not restricted to the plasma membrane, which allows the aggregation of most membrane structures cross-linked to the cytoskeleton. This concept will hopefully advance the study of OC biology centered around the cytoskeleton and membrane proteins.

In this review, we present the structure and function of the membrane microdomains in OCs (Table 4). We selected prominent membrane microdomain structures, including lamellipodia, TNTs, ZLSs, and secretory lysosomes, based on the most critical aspects of OC biological behavior: multinucleation and bone resorption processes. Other membrane microdomains, such as the caveolar structure, which affects endocytosis, or reggie proteins, which may be involved in early fusion, also require further research. Additionally, other cells may exhibit the same membrane microdomains as OCs. Lamellipodia are involved not only in epithelium-associated cell migration but also possibly in the formation of lateral dendritic branches and myelin sheaths.^57,113–115^ Additionally, the secretory lysosomes of OCs are a specific type of LRO, and LROs mediate endoplasmic reticulum-associated protein degradation.^116^ In addition, in neurons, membrane fusion with autophagosomes may be triggered when LROs are specifically modified. These findings suggest that the practical applications of membrane microdomains may have a markedly wider range than those summarized above. We hypothesize that the commonalities and identities of membrane microdomains can be categorized by comparing and combining them among models. Therefore, we expect to identify the crucial scaffolding proteins that determine the function and formation of the membrane. This approach may allow us to obtain new insights into the core proteins in TNTs and ZLSs that are currently uncharacterized.

Moreover, some core membrane proteins and their related signaling mechanisms remain incompletely understood. The cross-linking between scaffolding proteins and regulation of the actin cytoskeleton via intra- and extracellular signaling is also poorly understood. These functions need to be further characterized in subsequent studies. In conclusion, this review summarizes the membrane microdomains at different stages of the OC life cycle to provide a reference for studying membrane microdomain-targeted therapies that selectively inhibit OCs at different phases and can be used to treat OC-related metabolic bone diseases.
